# The microbiome in pediatric cystic fibrosis patients: the role of shared environment suggests a window of intervention

**DOI:** 10.1186/2049-2618-2-14

**Published:** 2014-04-28

**Authors:** Thomas H Hampton, Deanna M Green, Garry R Cutting, Hilary G Morrison, Mitchell L Sogin, Alex H Gifford, Bruce A Stanton, George A O’Toole

**Affiliations:** 1 Department of Microbiology and Immunology, Geisel School of Medicine at Dartmouth, 615 Remsen Building, N. College St., Hanover, NH 03755, USA; 2 Division of Pediatric Pulmonary and Sleep Medicine, Duke University Medical Center, 350 Hanes House, DUMC Box 102360, Durham NC, USA; 3 McKusick-Nathans Institute of Genetic Medicine, Johns Hopkins University School of Medicine, 1800 Orleans St., Baltimore MD, USA; 4 Josephine Bay Paul Center for Comparative Molecular Biology and Evolution, Marine Biological Laboratory, 7 MBL Street, Woods Hole, MA 02543, USA; 5 Department of Microbiology and Immunology, Geisel School of Medicine at Dartmouth, 202 Remsen Building, N. College St., Hanover, NH 03755, USA

**Keywords:** Cystic fibrosis, Microbiome, *Pseudomonas aeruginosa*, Sputum

## Abstract

**Background:**

Cystic fibrosis (CF) is caused by mutations in the *CFTR* gene that predispose the airway to infection. Chronic infection by pathogens such as *Pseudomonas aeruginosa* leads to inflammation that gradually degrades lung function, resulting in morbidity and early mortality. In a previous study of CF monozygotic twins, we demonstrate that genetic modifiers significantly affect the establishment of persistent *P. aeruginosa* colonization in CF. Recognizing that bacteria other than *P. aeruginosa* contribute to the CF microbiome and associated pathology, we used deep sequencing of sputum from pediatric monozygotic twins and nontwin siblings with CF to characterize pediatric bacterial communities and the role that genetics plays in their evolution.

**Findings:**

We found that the microbial communities in sputum from pediatric patients living together were much more alike than those from pediatric individuals living apart, regardless of whether samples were taken from monozygous twins or from nontwin CF siblings living together, which we used as a proxy for dizygous twins. In contrast, adult communities were comparatively monolithic and much less diverse than the microbiome of pediatric patients.

**Conclusion:**

Taken together, these data and other recent studies suggest that as patients age, the CF microbiome becomes less diverse, more refractory to treatment and dominated by mucoid *P. aeruginosa*, as well as being associated with accelerated pulmonary decline. Our studies show that the microbiome of pediatric patients is susceptible to environmental influences, suggesting that interventions to preserve the community structure found in young CF patients might be possible, perhaps slowing disease progression.

## Findings

### Background

Cystic fibrosis (CF) is an inherited disease that affects over 70,000 people worldwide. It is caused by mutations in the CF transmembrane conductance regulator (*CFTR*) gene. Mutations in *CFTR* reduce airway surface liquid volume, thereby reducing mucociliary clearance of bacterial pathogens that cause chronic inflammation [1]. Chronic infection results in a decline of respiratory function and eventual pulmonary failure, despite aggressive and repeated use of antibiotics. Although the overall process of disease progression is unfortunately predictable, individual rates of progression can vary tremendously, leaving some CF patients relatively healthy in their 30s and others in need of lung transplants in their teens [2].

*Pseudomonas aeruginosa* is an opportunistic pathogen found in approximately 80% of adult CF patients. It is the presumed cause of most pulmonary exacerbations and therefore the major target of antibiotic treatment in CF patients [3]. Culture-dependent techniques applied to the study of CF sputum are used to identify antibiotic-resistant and susceptible strains of *P. aeruginosa*, as well as *Haemophilus influenzae*, *Burkholderia cepacia* complex, *Staphylococcus aureus*, *Stenotrophomonas maltophilia* and methicillin-resistant *S. aureus*, which also contribute to the etiology of the CF phenotype [4]. Culture-independent techniques (for example, 454 pyrosequencing of the bacterial 16S rRNA gene) can identify over 100 distinct genera, including the commensal genera that provide the microbial diversity found in children that gradually disappears in adults. The results of studies such as those published by Cox *et al*. [5] and Madan *et al*. [6] show that the diversity of the CF microbiome peaks during childhood, then falls during the second decade of life, when it is more frequently dominated by *P. aeruginosa*. Goddard *et al*. [7] showed that end-stage CF is associated with reduced community diversity and domination by a few antibiotic-resistant pathogens such as *P. aeruginosa* and *B. cepacia* complex.

Investigators in a recent study of CF twins found that pulmonary function among monozygous (MZ) twins is more similar than that between dizygous (DZ) twin and sibling pairs, regardless of whether they live together or apart [8], suggesting that genetic modifiers play significant roles in CF pulmonary function. In their study, genetic factors accounted for 50% of pulmonary function variation, and, collectively, the remaining 50% were explained by both stochastic and deterministic environmental influences. Specifically, unique environmental or stochastic factors accounted for 36%, and shared environmental factors accounted for 14% of the remaining variation. Infection with environmental pathogens such as *P. aeruginosa* contributes substantially to the progression of lung disease in CF [9-11]; thus it is reasonable to expect that genetic and/or nongenetic modifiers also impact infections. Indeed, the timing of development of chronic *P. aeruginosa* infection was more similar in MZ twins than in DZ twins or other siblings, suggesting a strong genetic influence of this trait [12].

In this study, we hypothesized that environmental factors substantially influence the development of the CF pediatric microbiome, suggesting the possibility of manipulating these communities to a more healthful state. We provide new insight into the mechanisms by which shared environment contributes to infection status in CF patients.

## Methods

### Patient consent and ethical approval

Adults who were 18 years of age or older with CF confirmed by genotype analysis were recruited from the CF program at Dartmouth-Hitchcock Medical Center (DHMC). They provided written informed consent as approved by the DHMC Institutional Review Board. Children ages 10 to 18 years old with CF confirmed by genotype analysis were recruited from the CF program at Johns Hopkins University School of Medicine. They provided written informed consent as approved by the Johns Hopkins University School Institutional Review Board.

### Data collection and statistical analysis

We analyzed the microbiome in the sputum from thirteen clinically stable pediatric siblings from six families, of which eight siblings were MZ twins and five were siblings who served as proxies for DZ twins because they differed in age by less than 3 years from the other child with CF living in the same household. We compared the sputum microbiomes of these pediatric patients (median age: 14 years) with each other and also to those of ten clinically stable adults with CF chosen as randomly assigned controls in an adult CF microbiome study (median age: 30 years) living apart [13]. The detailed demographics of these samples can be found in Additional file 1: Tables S1 and S2. Pediatric samples were collected in accordance with induced sputum protocols [14], and adult samples were spontaneously produced as described by Gifford *et al.*[13]. As CF twins are rare, our CF pediatric samples were collected at seven different regional CF centers from Texas to New York. Adult samples were collected at DHMC. DNA isolated from the resulting 43 samples was deep-sequenced, and unique genera were identified on the basis of variable regions of the bacterial 16S ribosomal gene as previously reported [15]. Three replicate samples from adults were averaged to form ten representative samples. The sequence reads were similar between sets, with an average of approximately 15,000 reads per pediatric sample and about 12,000 reads per adult sample. Bray-Curtis dissimilarity and Shannon Diversity Index were measured using the ecodist [16] and vegan [17] packages in R statistical software. Significant differences between groups were established using analysis of variance with Tukey’s Honest Significant Difference posttest. Tests producing a *P* value less than 0.05 were deemed significant. The pediatric data sets have been deposited in the Sequence Read Archive database (NCBI BioProject PRJNA237382; National Center for Biotechnology Information (NCBI), US National Library of Medicine, Bethesda, MD, USA), and all data sets are also available at http://vamps.mbl.edu/ (Visualization and Analysis of Microbial Population Structures (VAMPS), Josephine Bay Paul Center, Woods Hole, MA, USA) [18]. A summary of the sequencing data read numbers and fractions can be found in the supplemental data (Additional file 2).

## Results

**Figure 1:**
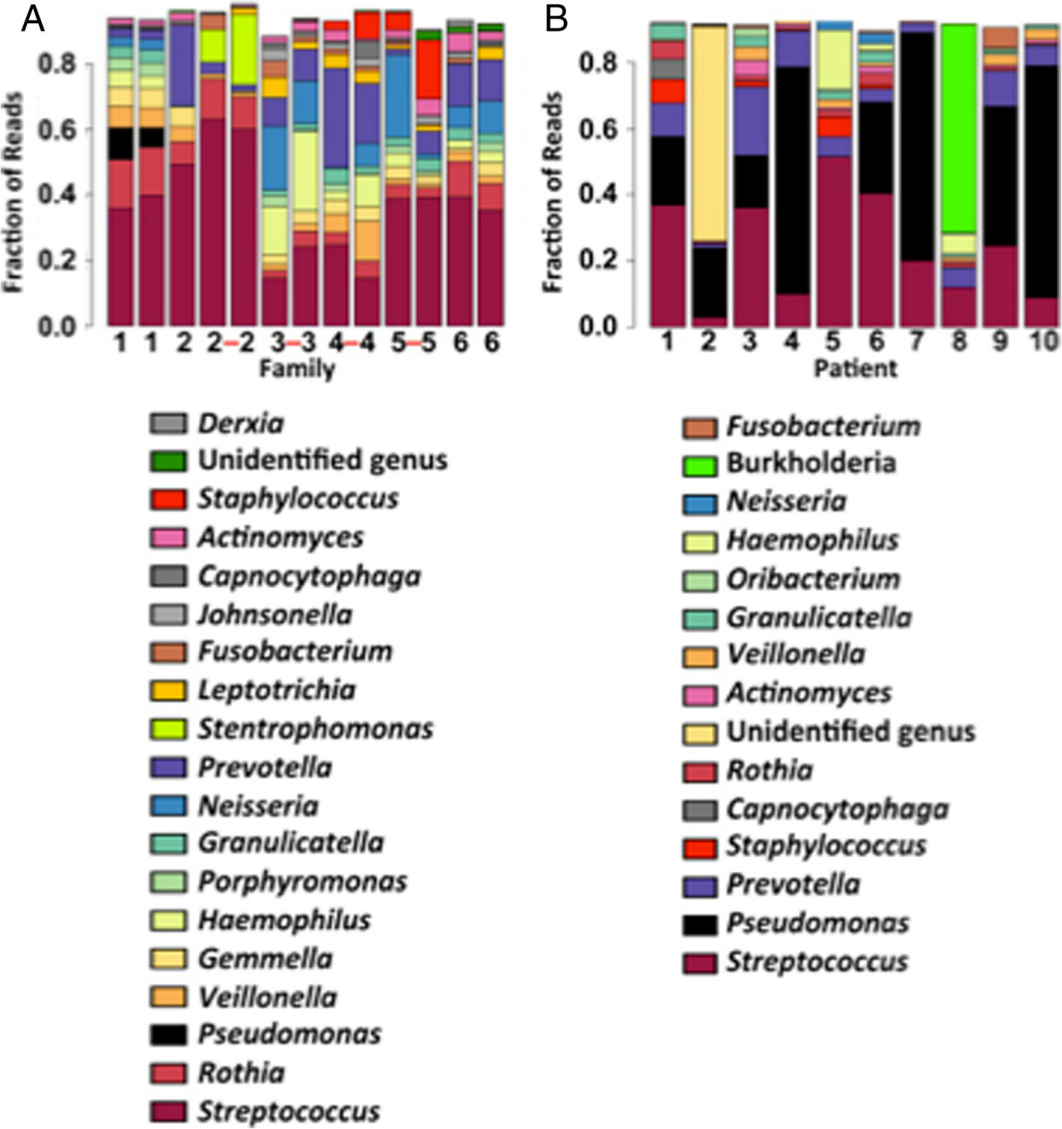
**Analysis of microbial communities in pediatric and adult patients. (A)** Nineteen genera accounted for 85% of the reads in sputum collected from thirteen pediatric patients with cystic fibrosis (CF). Data are presented in a stacked bar chart of relative abundance as fractions of total reads (*y*-axis) for the top 19 genera for each patient (*x*-axis). Families are numbered 1 to 6, and pairs of monozygous twins are connected with a red dash. The color key indicating these 19 genera is shown at the bottom of the figure. Pediatric samples contain a large fraction of genus *Streptococcus*, shown at the bottom of the bars in maroon. **(B)** Fifteen genera accounted for 85% of the reads in sputum collected from ten adult patients with CF. Adult samples are often dominated by the genus *Pseudomonas*, shown in black. *Burkholderia*, shown in green, is dominant in patient 8.

**Figure 2:**
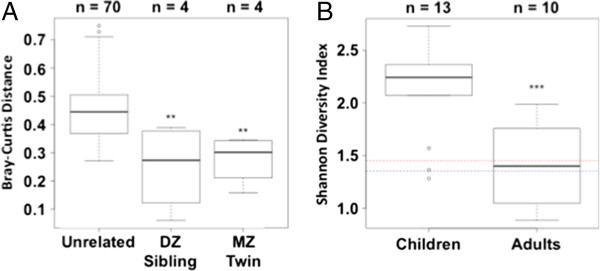
**Relatedness and diversity of microbial communities. (A)** Box-and-whisper plots of pairwise Bray-Curtis distances of the microbiomes of pediatric cystic fibrosis (CF) patients as a function of genetic relatedness, showing significantly more distance between unrelated patients than between related patients (*P* = 0.01), but no significant difference in relatedness between monozygotic (MZ) twins and nontwin siblings with CF (a proxy for dizygotic (DZ) twins, labeled “DZ Sibling”). Boxes indicate median values as the center line of each box, which spans the interquartile range. Whiskers demarcate 1.5× the interquartile range. These values reflect 70 pairwise comparisons among the samples from the ten unrelated subjects, and four pairwise comparisons among samples from the five siblings. **(B)** Boxplots comparing Shannon Diversity Index in all children with CF in this study and adults with CF reveal significantly lower diversity in adult versus the pediatric cohort (*P* = 0.001). Median Shannon Diversity Index value from adult patients in the Tunney *et al*. study [19] is shown as the red line, and the median Shannon Diversity Index score in adult patients reported by Zhao *et al*. [20] is shown as a blue line for comparison to our adult cohort. Whiskers denote 1.5 times the interquartile range defined by the box. Open circles denote outlier values outside this range. Significance codes: ‘***’ P < 0.001; ‘**’ P < 0.01.

Figure 1A shows the fraction of reads and associated bacterial genera for each of our 13 pediatric patients from 6 different families. Notably, the microbiomes of these pediatric patients is dominated by *Streptococcus* spp., and *P. aeruginosa* accounted for a small fraction of reads in any given sample. Comparing siblings living together to unrelated individuals reveals that the environment plays an important role in determining the composition of the microbiota. Both identical twins and siblings living together (twins connected by a red dash in Figure 1A) have very similar communities. For example, siblings in families 1 and 6 are much more like each other than they are to children from other families (Figure 1A). We also compared the microbiomes of pediatric CF patients using Bray-Curtis dissimilarity analysis (Figure 2A), which confirmed that the siblings from the same family are significantly more similar to each other than they are to unrelated pediatric CF patients. Importantly, there was no significant difference in relatedness of communities between MZ twins versus nontwin siblings with CF (a proxy for DZ twins) living together, despite the genetic differences in these cohorts. This finding supports the hypothesis that the environment may influence the airway community.

Figure 1B shows the fraction of reads and associated bacterial genera for ten unrelated adult patients. In contrast to the pediatric CF patient cohort, the microbiomes of adult CF patients are dominated by *P. aeruginosa*, with relatively little *Streptococcus*. In addition, a visual inspection of the communities in the pediatric and adult CF cohorts indicated that microbiome diversity is higher in the pediatric cohort than in the adult cohort (compare Figure 1A with Figure 1B). This observation was confirmed by analysis of diversity using the Shannon Diversity Index (Figure 2B) and is consistent with previous work showing that the biomes of infants with CF are dominated by *Veillonella* and *Streptococcus*. Diversity rapidly increases in the first 21 months of life [6] and peaks at around 11 years of age [21].

## Conclusions

Our analysis of the sputum microbiomes in pediatric CF patients supports the conclusion that environmental factors—that is, all manner of nongenetic factors—play an important role in determining the composition of the CF microbiome and is in agreement with the results of other studies of adults with CF that the environment is an important determinant of the microbiomes found in these patients [22]. We found that the microbiomes of pediatric CF patients living together are much more alike than those among individuals living apart, and, importantly, there was no significant difference between affected MZ twins and affected siblings living together. These observations are consistent with the view that the environment is an important factor in determining the composition of the microbiome. Although it is possible that a much larger sample size might resolve subtle differences between affected siblings living together and affected MZ twins living together, we saw no such difference in our small study. Nonetheless, we could easily see differences between pediatric patients living in the same house compared to those living apart. In any case, numerous studies have identified an inverse relationship between microbial diversity and lung function, as well as inflammatory markers, in airway samples of adults [23,24]. These observations suggest that novel therapeutic regimens that maintain diversity while reducing total bacterial load may improve clinical outcomes and prevent the development of a microbiome dominated by mucoid *P. aeruginosa* that is tolerant to antibiotic therapy. It is also possible that early exposure to microbes and allergens permanently shapes the developing microbial community in the lungs of CF patients. Such imprinting could mean that patients who live together are exposed to, and therefore colonized by, the same microbes. It would be interesting to know whether the microbiota of these pediatric CF patients living together would begin to diverge when they are removed from the same home environment.

Importantly, the observation described herein that the pediatric microbiome can be influenced by environment suggests that the microbial communities in these patients might be amenable to manipulation. We hypothesize that early intervention to enhance or maintain the diversity of the microbiota of young CF patients, perhaps by using probiotics, holds promise for slowing disease progression until a cure is found for this pernicious disease. Although our data suggest that maintaining pediatric microbial diversity into adulthood may lead to better outcomes, effective strategies to maintain diversity will require a clearer understanding of how microbial communities in the CF pediatric airway evolve under the selective pressure of repeated antibiotic treatment.

## Availability of supporting data

The data sets supporting the results of this article are included within the article (and its additional files).

The data sets supporting the results of this article are available in the SRA repository, http://www.ncbi.nlm.nih.gov/sra/?term=SRP038106.

## Abbreviations

CF: Cystic fibrosis; DZ: Dizygous; MZ: Monozygous.

## Competing interests

The authors declare that they have no competing interests.

## Authors’ contributions

THH performed statistical analyses and manuscript writing. DMG, HGM and AHG performed data collection and analysis and manuscript writing. GRC, BAS and GAO were responsible for the conception and design of the study and securing financial support, and participated in writing the manuscript. MLS performed data collection and analysis and secured financial support, and participated in writing the manuscript. All authors read and approved the final manuscript.

## Supplementary Material

Additional file 1: Table S1 and Table S2 Two supplemental tables summarizing the patient data for the patient cohorts that were the subject of the study. CF, Cystic fibrosis; MaxFEV_1_, Maximum volume exhaled at end of first second of forced expiration. Click here for file

Additional file 2 **Sequence data.** Summary of demographic data of each sample analyzed, as well the number and fraction of reads from adult and pediatric samples. Click here for file
