# Supplementary material for: The microbiome in pediatric cystic fibrosis patients: the role of shared environment suggests a window of intervention
Source: Microbiome. 2014 Apr 28;2:14. doi: 10.1186/2049-2618-2-14 (PMC4113139; doi:10.1186/2049-2618-2-14)
Supplement: Additional file 1: Table S1 and Table S2 — Two supplemental tables summarizing the patient data for the patient cohorts that were the subject of the study. CF, Cystic fibrosis; MaxFEV1, Maximum volume exhaled at end of first second of forced expiration. [file 2049-2618-2-14-S1.pdf]

Supplemental Table 1: Characteristics of study population by twin/sibling status

	MZ twins	Siblings
Individuals	8	5
Female gender	2 (25%)	0
Caucasian	8 (100%)	5 (100%)
CF genotype		
ΔF508 homozygotes	4 (50%)	5 (100%)
Pancreatic insufficient	8 (100%)	5 (100%)
Age ± SD, in yrs (range)	14.25 ± 2.97 (10.98-18.08)	13.76 ± 2.41 (10.29-16.10)
$MaxFEV_1$ CF% ile	0.71 ± 0.28 (0.191-0.976)	0.78 ± 0.19 (0.58-1.0)
Previous positive *P. aeruginosa* culture	8 (100%)	5 (100%)
Previous positive *S. aureus* culture	8 (100%)	5 (100%)

Supplemental Table 2: Characteristics of adult study population

Individuals	10
Female gender	2 (20%)
Caucasian	10 (100%)
CF genotype	
ΔF508 homozygotes	6 (60%)
Pancreatic insufficient	10 (100%)
Age ± SD, in yrs (range)	35.4 ± 15.3 (20.1-50.7)
$MaxFEV_1$ CF% ile	0.57 ± 0.21 (0.36-0.78)
Previous positive *P. aeruginosa* culture	10 (100%)
Previous positive *S. aureus* culture	10 (100%)
